# Development of a Cre-Inducible Rabl6a Transgenic Mouse Model That Enhances Sarcoma Growth In Vivo

**DOI:** 10.3390/cancers18142230

**Published:** 2026-07-11

**Authors:** Ellen M. Voigt, Alexandra L. Isaacson, Mariah R. Leidinger, James A. Goeken, Quinn Hanigan, Deng Fu Guo, Rachel M. Gasser, Makenna Eadie, Isabella Babor, Benjamin W. Darbro, William Paradee, Kamal Rahmouni, Tian Zhao, Patrick Breheny, Eunhyeong Lee, Minah Kim, David K. Meyerholz, Mohammed Milhem, Rebecca D. Dodd, Dawn E. Quelle

**Affiliations:** 1 Cancer Biology Graduate Program, University of Iowa, Iowa City, IA 52242, USA; ellen-voigt@uiowa.edu (E.M.V.);; 2 Holden Comprehensive Cancer Center, University of Iowa, Iowa City, IA 52242, USA; 3 Medical Scientist Training Program, Carver College of Medicine, University of Iowa, Iowa City, IA 52242, USA; 4 Department of Pathology, Carver College of Medicine, University of Iowa, Iowa City, IA 52242, USA; 5 Department of Neuroscience and Pharmacology, Carver College of Medicine, University of Iowa, 2-570 Bowen Science Bldg., 51 Newton Rd, Iowa City, IA 52242, USA; 6 Department of Internal Medicine, Carver College of Medicine, University of Iowa, Iowa City, IA 52242, USA; 7 Department of Pediatrics, Carver College of Medicine, University of Iowa, Iowa City, IA 52242, USA; 8 Genome Editing Core Facility, Carver College of Medicine, University of Iowa, Iowa City, IA 52242, USA; 9 Iowa City Veterans Affairs Health Care System, Iowa City, IA 52242, USA; 10 Department of Biostatistics, College of Public Health, University of Iowa, Iowa City, IA 52242, USA; 11 Department of Pathology and Cell Biology, Irving Medical Center, Columbia University, New York, NY 10032, USA

**Keywords:** Rabl6a, MPNST, sarcoma, Cre, Desert hedgehog (Dhh), rhabdomyoblastic differentiation (RMB)

## Abstract

**Background:** Malignant peripheral nerve sheath tumors (MPNSTs) are deadly sarcomas that arise from Schwann cells and lack effective therapies. RABL6A is an oncogenic Rab-like GTPase whose expression is associated with worse survival in many human cancers. It is required for human MPNST cell survival, and its expression is dramatically increased in patient MPNSTs compared to benign precursor lesions. **Methods:** To model elevated expression of RABL6A in vivo, we developed transgenic mice expressing Cre-inducible Rabl6a. These Rabl6a-tg mice express the murine Rabl6a cDNA with a 5′ hemagglutinin [HA] epitope sequence downstream of a CMV enhancer and separated by a lox–stop–lox cassette. Double transgenic DhhCre; Rabl6a-tg mice were generated to achieve Schwann-cell specific Cre expression from the Desert hedgehog (Dhh) promoter. De novo MPNSTs were induced by CRISPR editing of *Nf1*, *Ink4a*, and *Arf* genes in the mouse sciatic nerve. **Results:** Cre-dependent expression of transgenic Rabl6a was verified at the mRNA and protein levels in Cre-positive mouse embryo fibroblasts and tissues. Increased Rabl6a expression in DhhCre; Rabl6a-tg mice had no effect on de novo MPNST initiation but significantly accelerated tumor progression relative to DhhCre control mice. The Rabl6a phenotype was associated with increased tumor angiogenesis but not proliferation. Interestingly, many MPNSTs in the DhhCre background exhibited varying levels of rhabdomyoblastic (RMB) features. That immature muscle cell phenotype is a hallmark of malignant Triton tumors, a rare histological variant of human MPNSTs associated with worse outcomes. **Conclusions:** These data provide direct evidence that Rabl6a is a functional driver of MPNSTs while establishing Rabl6a-tg mice as a suitable model for investigating Rabl6a’s role in other lethal RABL6A-high tumors.

## Simple Summary

This work examined the in vivo importance of elevated RABL6A, an oncoprotein that is overexpressed in many human tumors, in a type of sarcoma called malignant peripheral nerve sheath tumors (MPNSTs). Patient MPNSTs express high levels of RABL6A. Therefore, new transgenic mice were developed that express Cre-inducible, murine Rabl6a in Schwann cells, the originating cell that can transform into MPNSTs. When MPNSTs were generated in these mice, Schwann cell-specific expression of Rabl6a caused tumors to grow more quickly. Intriguingly, many MPNSTs in these mice displayed immature muscle cell differentiation, which mimics a rare, aggressive subtype of human MPNST that is poorly understood. This work shows that Rabl6a upregulation drives MPNST progression while offering a new mouse model for studying other RABL6A-high cancers where its expression worsens patient outcomes.

## 1. Introduction

Malignant peripheral nerve sheath tumors (MPNSTs) are aggressive and painful sarcomas that develop due to Schwann cell transformation in the nerve sheath [1]. Half of these tumors arise spontaneously while the other half are associated with a relatively rare genetic disease called Neurofibromatosis Type I (NF1) [2]. In NF1 patients, the tumor originates as a benign plexiform neurofibroma (PNF) that over time can acquire more mutations, resulting in a more advanced benign lesion termed an Atypical Neurofibromatous Neoplasm of Uncertain Biological Potential or ANNUBP [3,4]. Up to 13% of the time, these benign lesions can transform into MPNSTs, which are the leading cause of death for NF1 patients, particularly young adults [1]. Currently, the only curative option for MPNSTs is complete surgical resection with clear negative margins, which, depending on the location and infiltration of the tumor, can be impossible [2,5]. Chemotherapy and radiation are used to achieve local control of MPNSTs, but these treatments do not increase the dismal five-year survival rate of 50–60% [5,6]. MPNST patients desperately need new treatments. Thus, there is a critical need to understand the key molecular alterations driving MPNST transformation since that knowledge should guide the development of new targeted therapies [7,8].

Some molecular changes that drive MPNST formation are well described. These include loss of heterozygosity of the *NF1* gene, an initiating event for PNF formation that is also seen in all sporadic MPNSTs, and loss of the *CDKN2A* tumor suppressor locus [3,4,9]. *CDKN2A* encodes two unrelated tumor suppressor proteins, p16INK4a and p14ARF [10], whose deletion or mutation is found in the vast majority of NF1-associated and sporadic MPNSTs. Its loss in NF1 tumors is a defining marker of the intermediate ANNUBP stage. However, the transition to an MPNST remains incompletely understood and is characterized by marked heterogeneity in genetic and molecular changes that include the loss of *SUZ12*, *EED*, and *TP53* [11,12]. It is recognized that many other molecular changes occur in MPNSTs. A high priority in the field is to better study all the different individual alterations to better understand this tumor [7].

RABL6A is a relatively understudied oncoprotein that our lab found is significantly upregulated in human MPNSTs relative to benign PNFs and ANNUBPs [13]. RABL6A (Rab-like isoform 6A) is a large GTPase that was first discovered as a binding partner of the ARF tumor suppressor (p14ARF in human, p19ARF in mice) [14]. In the following years, RABL6A was identified as a new member of the Ras/Rab superfamily through in silico screening, found to be overexpressed in breast and colon cancer cell lines, and shown to be required for their proliferation and survival [15,16]. Several names for RABL6A exist, including PARF (Partner of ARF), RBEL1A (Rab-like protein 1), and c9orf86. To date, elevated levels of RABL6A in patient tumors have been associated with poor survival and/or metastasis for multiple cancers including esophagus squamous cell carcinoma, breast cancer, pancreatic ductal adenocarcinoma, various types of sarcoma including MPNST and osteosarcoma, hepatocellular carcinoma, and non-small-cell lung cancer [17,18,19,20,21,22,23]. We and others have shown that RABL6A promotes tumorigenesis through many pathways such as AKT activation via PP2A inhibition, Mdm2-mediated degradation of p53, activated MEK-ERK signaling, and CDK4/6-mediated inactivation of RB1 [13,20,24,25,26,27]. A key role for RABL6A in MPNST biology was established using in vitro and in vivo approaches. Knockdown of RABL6A in human MPNST cells showed that it is required for their proliferation and survival [13], while global genetic deletion of endogenous *Rabl6a* in mice significantly slowed the progression of de novo MPNSTs [24].

To determine the impact of increased RABL6A on MPNST pathogenesis in vivo, thereby mimicking what is observed in patient tumors [13], we generated a conditional transgenic mouse model for controlled, inducible Rabl6a expression. The model relies upon Cre recombinase to overexpress an HA-tagged Rabl6a protein, enabling tissue- and time-dependent control of its expression with different Cre drivers. We describe the validation of this model and provide the first direct evidence that Schwann cell-specific expression of Rabl6a promotes de novo MPNST progression in vivo. This transgenic mouse provides a new system for in-depth investigations of Rabl6a function in MPNST biology; importantly, it also offers a platform for crosses with different tissue-specific Cre mice to study other RABL6A-high cancers where its expression portends poor patient survival, such as lung, liver, and breast cancers.

## 2. Materials and Methods

### 2.1. Mouse Model Generation

All mouse studies adhered to protocols approved by the Institutional Animal Care and Use Committees at the University of Iowa (protocol #7112074: Targeting RB1 Pathway in Sarcomas). Mice were housed at the University of Iowa Barrier facility in a climate-controlled room, with a 12 h light–dark cycle, and continual access to food and water. The Rabl6a-tg mouse model was created in collaboration with the Genomic Editing Facility at the University of Iowa. The transgene was targeted to the *Hipp11* safe locus but integrated randomly into the genome via CRISPR editing of mouse oocytes.

### 2.2. Mouse Embryonic Fibroblasts

To make mouse embryo fibroblasts (MEFs), Rabl6a-tg (R6A) mice were bred to Rosa26CreER (R26-CreER) mice (B6.129-Gt(ROSA)26Sortm1(cre/ERT2)Tyj/J>). The mice were housed together for one day and the dam’s weight was recorded. At 13.5 days after mating, if the dam’s weight had increased by at least 4 g, she was euthanized and her embryos were harvested. Embryos were removed from their sacs, their heads and internal organs were removed, and the rest of the tissue was homogenized mechanically with a razor blade and then enzymatically digested by incubation in 0.05% trypsin for 15 min. The resulting MEFs were plated in tissue culture flasks and fed with DMEM supplemented with 10% FBS, 2 mM glutamine, 10% penicillin/streptomycin, 55 µM 2-mercaptoethanol and 0.1 µM non-essential amino acids. To induce transgene expression, the cultured cells were treated with 1 µM 4-hydroxytamoxifen for 3 days (catalog #H7904, Sigma-Aldrich, St. Louis, MO, USA).

### 2.3. Long-Read Whole-Genome Sequencing

Genomic DNA from R6A MEFs was subjected to Oxford Nanopore long-read whole-genome sequencing through Plasmidsaurus (Louisville, KY, USA). Sequencing produced 13,120,011 reads totaling approximately 112.0 Gb, with a read-length N50 of 16,382 bp and a maximum read length of 414,025 bp—corresponding to approximately 40× coverage of the ~2.81 Gb mouse genome. Raw reads were length- and quality-filtered with nanoq v0.10.0 (minimum read length 1000 bp; minimum mean quality Q7). Filtered reads were assembled de novo with hifiasm v0.25.0 in Nanopore mode with parameters selected for high-quality ONT reads. The resulting primary assembly comprised 278 contigs spanning 2808 Mb with a contig N50 of 129.4 Mb, indicating a near-chromosome-scale assembly. The integration construct sequence was obtained from the SnapGene-format (.dna) map of the targeting vector (pHipp11_CAG-Rabl6_bGHpA; 10,177 bp). The .dna file was parsed with Biopython v1.87, and the integrated cassette, the 6272 bp sequence between the inner boundaries of the two homology arms, was exported in FASTA format for use as an alignment query. The cassette query was aligned to the complete de novo assembly with minimap2 [v2.26] (preset asm5) [28]. All alignments mapped to a single 107,638 bp contig (ptg000121l), to which the full-length cassette aligned at five distinct positions (≥99.5% identity), identifying ptg000121l as the transgene-bearing contig and indicating multi-copy integration.

### 2.4. Characterization of the Integration Locus in the Mouse Genome

To resolve copy number, orientation, and internal structure, the cassette, the 5′ and 3′ H11 homology arms, the bacterial backbone, and the loxP-flanked stop block were each aligned to ptg000121l with minimap2 (asm5, reporting secondary alignments). Copy number, repeat-unit composition, orientation, and array extent were inferred from the genomic coordinates and strand of the resulting alignments. The contig segments flanking the transgene array (the regions of ptg000121l lacking any alignment to the construct) were extracted and aligned to the mouse reference genome (GRCm39/mm39) using BLAT (UCSC Genome Browser) [29,30]. The reference coordinates immediately abutting the array on each side were taken as the transgene–genome junctions, and if a host sequence was absent between the two junctions it was scored as a target-site deletion. To confirm the integration locus independently of the de novo assembly, the quality-filtered reads were mapped to a hybrid reference consisting of the mouse reference genome (GRCm39/mm39) concatenated with the extracted cassette sequence, using minimap2 [v2.26] (preset map-ont); alignments were coordinate-sorted and indexed with samtools [v1.12] [31]. Reads with any alignment to the cassette contig were identified, and the genomic distribution of all of their primary and supplementary alignments was tabulated by chromosome. Reads spanning chromosome 10 junctions were further examined in IGV [v2.19.1] [32] with soft-clipped bases displayed and supplementary alignments linked, and the supplementary-alignment (SA) tags of individual junction-spanning reads were inspected to confirm that single molecules crossed from a unique chromosome 10 sequence into the transgene array.

### 2.5. Tamoxifen Treatment of Rabl6a-tg–Rosa26CreER Mice and Controls

R6A and R26-CreER mice were bred together to characterize in vivo tissue expression of Rabl6a. When offspring of that cross were 16–24 weeks old, half of them were injected with 75 mg/kg tamoxifen (TAM, Sigma-Aldrich T5648, dissolved in sunflower oil, Sigma-Aldrich S5007) by i.p. for 5 consecutive days, as described in [33]. All mice were sacrificed 3 weeks later, and their tissues were harvested for histology. Two mice of each genotype were harvested—one male and one female. For the analyses of DRGs, a larger study was performed that involved 9 R26-CreER; R6A mice treated with TAM (6 female and 3 male) compared to 8 untreated R26-CreER; R6A mice (4 female and 4 male) and 9 TAM-treated R26-CreER mice (5 female and 4 male). Mice underwent terminal transcardiac perfusion with 4% PFA to preserve their nervous systems. The spinal column was dissected, fixed for 2 days in 10% NBF, decalcified with 14% EDTA for 3 days, embedded in paraffin and sectioned. Multiple DRGs (2 to 8) were quantified per mouse.

### 2.6. De Novo MPNST Analyses in Rabl6a-tg–DhhCre Mice and Controls

Mice for this experiment were bred from crosses between Rabl6a-tg (R6A) mice and DhhCre mice (gift from Nancy Ratner, Cincinnati Children’s Hospital [34]), both on a C57BL/6N background. In these studies, 11 DhhCre mice (4 female and 7 male) and 14 DhhCre; R6A mice (8 female and 6 male) were examined. Tumors were generated through surgical exposure of the sciatic nerve and injection of an adenovirus carrying the CRISPR Cas-9 enzyme and guide RNAs for *Nf1*, *Ink4a*, and *Arf* [35]. Approximately 90 days later, an MPNST formed at that site in the mouse leg and was measured daily with electronic calipers until the tumor reached 2000 mm^3^ in volume, 2 cm in any dimension, or the mouse was too sick to continue. The tumor height, width and depth were measured and volume was calculated with the equation (H × W × D × π)/6. Tumor progression is represented as fold change, which was calculated as the volume of day/initial tumor volume.

### 2.7. PCR for Genotyping

For genotyping the Rabl6a-tg mice, several primers targeting the HA tag sequence were developed. The optimal primers (see Figure S1) employed a forward (5′—GGGATACCCATACGACGTACC—3′) and reverse (5′—CTCGACTTCGAGCCTCCATC—3′) pair that generated an 800 kb pair product. The PCR products were electrophoresed on a 1% agarose gel with ethidium bromide at 100 V for 30 min. Images of the products on gels were taken with a UVP Bioimaging system. Original images of the gels can be seen in Figure S5.

### 2.8. RT-qPCR in Tumors

Tumors were crushed using liquid nitrogen to freeze them and a mortar and pestle to mechanically grind them into a powder. RNA was extracted from the powder using a Qiagen RNeasy kit (Qiagen, Germantown, MD, USA, cat #74134) and cDNA was made using the SuperScript III First Strand cDNA prep kit (ThermoFisher, Waltham, MA, USA, cat #18080051). RT-qPCR was performed for the HA tag in the *Rabl6a* transgene (Fwd: 5′—CTGCAGAATTCAGGCCTACAC—3′, Rev: 5′—CCCACCAGCTTCTTCAACG—3′) and *Gapdh* (Fwd: 5′—GTTGTCTCCTGCGACTTCA—3′, Rev: 5′—GGTGGTCCAGGGTTTCTTA—3′) using iQ SYBR Green Supermix (Biorad, Hercules, CA, USA, cat# 1708880) on a Biorad CFX Opus 96 qPCR instrument using an annealing temperature of 56 °C. Expression of the HA tag was normalized to the *Gapdh* expression (ΔCT) and then to the HA expression of RNA extracted from a wild-type MEF cell line (ΔΔCT). Log2FC was calculated using 2^−ΔΔCT^. RT-qPCR products were run on a 1% agarose gel with ethidium bromide at 100 V for 30 min. An image of the RT-qPCR products was taken with a UVP Bioimaging system. Original images of the gels can be seen in Figure S5.

### 2.9. Western Blotting

MEF cell pellets were lysed in RIPA, protein was quantified by a Bradford assay and samples were boiled in SDS-PAGE buffer. Samples were run in polyacrylamide gels, transferred onto a PVDF membrane, and blocked with 5% BSA in TBST buffer for 1 h. Membranes were incubated in primary antibody overnight. The primary antibodies used were Gapdh (1:1000 in 5% milk in TBST, Abcam, Waltham, MA, USA, cat #ab8245) and Rabl6a 2N (5 μg/μL in 5% BSA in TBST, Quelle Lab [14]). The membranes were washed, incubated in HRP-conjugated secondary antibody in 5% milk in TBST, soaked in enhanced chemiluminescence reagent (ECL, Amersham, Buckinghamshire, UK), and images were captured with film. Signal was quantified with ImageJ version 1.54t. Original images of the blots can be seen in Figure S5.

### 2.10. Pathology

Tissue samples and tumors were harvested from euthanized mice, fixed (in 10% neutral buffered formalin) for at least 2 days and then embedded in paraffin. Tissue samples were cut and subjected to hematoxylin and eosin (H&E) staining by the Comparative Pathology Laboratory (University of Iowa). Immunohistochemistry (IHC) staining for the HA tag (1:1000, Cell Signaling Technologies, Danvers, MA, USA, cat #3724) in the HA-Rabl6a transgenic protein was performed on all tissue and tumors. For Ki67, IHC slides were stained with a mouse-on-mouse immunodetection kit (Vector Laboratories, Newark, CA, USA, BMK-2202) and a DAB substrate kit (Vector Laboratories, BMK-2202). Ki67 antibody was used at 1:500 (BD Pharmingen, San Diego, CA, USA, cat 556003). CD31 antibody was used at 1:50 (R&D Systems, Minneapolis, MN, USA, cat #AF3628). HA and Ki67 IHC slides were imaged at 10X BF on an Olympus Slideview VS200 and staining was quantified by counting the positive cells per field from three images per tumor using QuPath [36]. CD31 IHC slides were imaged at 20X BF on an Axio Observer.Z1/7. CD31 density was quantified by percent positive per area with ImageJ from the average of three images per tumor. All H&E was examined by a pathologist (ALI and/or DKM) to confirm presence of an MPNST and quantify the degree of rhabdomyoblastic (RMB) differentiation. Tumors from 13 DhhCre, 16 DhhCre, R6A and 14 WT C57BL/6N were examined. Numbers differ from the tumor kinetics experiment because some tumors were not measured over time but reached the size endpoint of the study and were evaluated for pathological findings. Ordinal scoring of tumor differentiation was performed following the principles of masking the pathologist to group assignment [37]. To distinguish RMB differentiation from myofiber regeneration at the invasive periphery of the tumor, the reviewing pathologist avoided classifying the region as RMB if it was adjacent to mature skeletal muscle.

### 2.11. Statistics

Quantified data were presented as the mean ± SEM. *p* values, unless otherwise specified, were obtained by One-way ANOVA and adjusted for multiple comparisons using the indicated method. Statistical analyses were performed using Prism GraphPad (San Diego, CA, USA) (α = 0.05). Overall differences between tumor growth rates were determined by comparing the slopes with a linear mixed model.

## 3. Results

### 3.1. Creation and Validation of the Rabl6a Transgenic (Rabl6a-tg) Mouse Model

**Figure 1:**
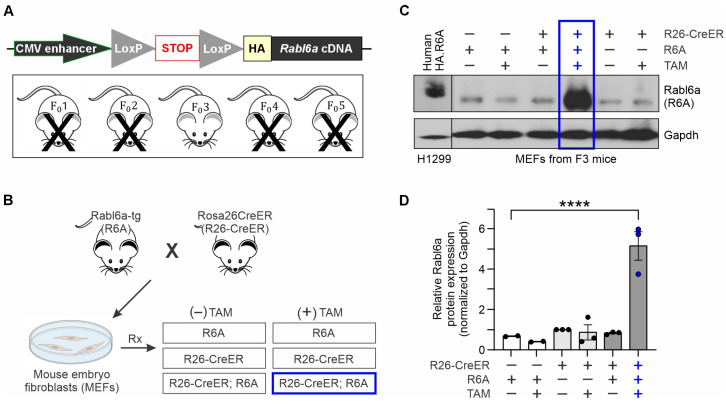
Generation and validation of a Rabl6a transgenic (Rabl6a-tg) mouse. (**A**) Simplified schematic of the *Rabl6a* transgene. The mouse Rabl6a cDNA was cloned with a 5′ HA sequence downstream of a lox–stop–lox (LoxP) site and a cytomegalovirus (CMV) early enhancer element within a CAG promoter. Five founder mice were created, but four were eliminated (marked by ‘X’) due to poor breeding or lack of transgene expression. Only founder 3 (F_0_3) consistently expressed Cre-inducible Rabl6a. (**B**) Overview of process for evaluating transgene expression. Mouse embryo fibroblasts (MEFs) were isolated from pregnant females (embryonic day 13.5) following crosses between Rabl6a-tg (R6A) and Rosa26CreER (R26-CreER) mice. Genotyping identified three distinct MEF populations (R6A, R26-CreER, or double-transgenic R26-CreER; R6A). MEFs were treated with vehicle (−) or tamoxifen (+ TAM) to specifically induce transgene expression only in R26-CreER; R6A cells (blue highlight). (**C**) Western blot of Rabl6a protein in lysates from the indicated MEF genotypes, with or without TAM treatment. The Rabl6a transgene was selectively induced in TAM-treated R26-CreER; R6A MEFs (blue highlight). H1299 cells expressing HA-tagged human RABL6A were used as a positive control. (**D**) Quantified data from two or three Western experiments showing reproducible induction of Rabl6a only in R26-CreER; R6A MEFs (blue highlight) treated with TAM relative to each control. ****, *p* < 0.0001.

The Rabl6a transgenic mouse was created by pronuclear injection of oocytes with CRISPR Cas-9 and the *Rabl6a* transgene to generate embryos with the inserted sequence. The transgene comprises a cytomegalovirus (CMV) enhancer, a LoxP–Stop–LoxP site, and a 5′ HA sequence that, when translated, will be in-frame with the mouse *Rabl6a* cDNA sequence (Figure 1A). These Rabl6a-tg mice should only express CMV-driven HA-Rabl6a when the LoxP sites and intervening stop codon are excised by Cre recombinase. Five founder mice were generated.

To validate conditional expression of the Rabl6a protein, each founder was bred to Rosa26CreER mice (enabling tamoxifen-induced expression of Cre recombinase in all tissues), and mouse embryonic fibroblast (MEF) cultures were generated from embryos isolated at E13.5 days (Figure 1B). Primary MEF cultures were either positive for the Rosa26CreER gene (abbreviated to R26-CreER), positive for the *Rabl6a* transgene (Rabl6a-tg, abbreviated to R6A), or double positive for both Rosa26CreER and the Rabl6a-tg (abbreviated to R26-CreER; R6A) (Figure 1B). Cells were then treated with vehicle control or tamoxifen (TAM) to induce Cre recombinase. Only double-positive R26-CreER; R6A MEFs treated with TAM are capable of overexpressing Rabl6a (blue box, Figure 1B). Lines from each founder were treated with or without TAM and the resulting levels of Rabl6a protein were measured by Western blotting (Figure 1C). Two founder mice were eliminated due to unsuccessful breeding while two others failed to effectively express the Rabl6a protein in double-positive, TAM-treated cells (see Figure 1A). Only founder line 3 (F_0_3) reproducibly expressed Cre-inducible Rabl6a in R26-CreER; R6A MEFs treated with TAM (blue box in Figure 1C, quantified in Figure 1D).

### 3.2. Characterization of the Rabl6a Transgene as a Multi-Copy Concatemer at a Single Intergenic Site on Mouse Chromosome 10

**Figure 2:**
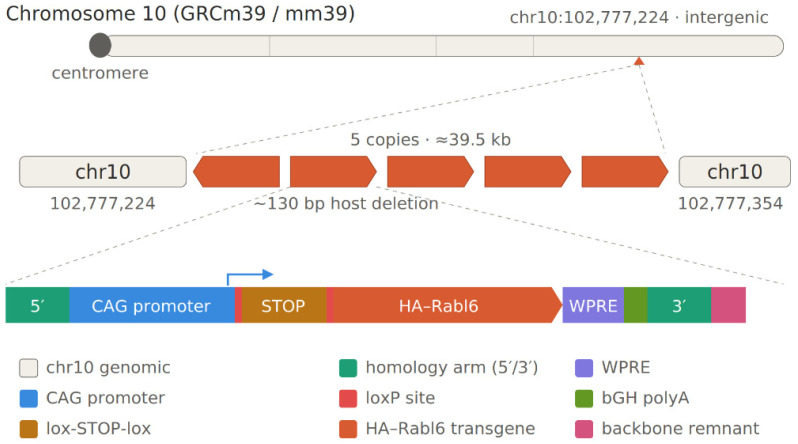
Insertion site of the *Rabl6a* transgene in the mouse genome. The transgene was inserted into a single site on chromosome 10 in a region devoid of genes (**top**). Five copies of the transgene were identified with four in the same orientation and the first insertion in reverse (**middle**). Each insertion was complete, with a 5′ homology arm intended for the *Hipp11* safe locus, a CAG promoter containing a CMV enhancer element, a stop codon, *HA-Rabl6a* cDNA, WPRE (Woodchuck Hepatitis Virus Posttranscriptional Regulatory Element), and bGH (bovine Growth Hormone) polyA regions, followed by the 3′ homology arm and a fragment of the vector backbone (**bottom**).

The *Rabl6a* transgene was intended to be inserted at the *Hipp11* (H11) safe harbor locus, but none of the founders had transgene integration at that site. To characterize where and how the transgene integrated into the genome, we performed whole-genome sequencing of *Rabl6a* transgenic MEFs, which yielded ~40× coverage with a read-length N50 of 16.4 kb (longest read 414 kb). De novo assembly produced a highly contiguous genome (278 contigs; contig N50 129.4 Mb; 2808 Mb total), providing near-chromosome-scale resolution and allowing the transgene insertion to be reconstructed within its native genomic context. The CAG–Rabl6 cassette localized exclusively to a single 107,638 bp contig (ptg000121l), within which it was present as five near-identical copies (≥99.5% nucleotide identity) arranged as an approximately 39.5 kb tandem array (Figure 2). Four copies were oriented head-to-tail, while the fifth, at the 5′end of the array, was inverted, forming a head-to-head junction (Figure 2). Each repeat unit consisted of a homology arm, the cassette, the second homology arm, and an ~340 bp backbone remnant (repeat period ~7.86 kb); the bulk of the bacterial backbone was absent. Because tandem repeats are prone to collapse during assembly, five copies represent a lower bound on the true copy number.

The genomic sequences flanking the transgene array were free of vector sequence and aligned to a single locus on mouse chromosome 10. The left and right flanks mapped to chr10: 102,777,224 and chr10: 102,777,354 (GRCm39), respectively, on the same strand and in the expected genomic order, defining a single integration site with an ~130 bp target-site deletion. The integration site lies within an intergenic region and does not disrupt an annotated gene. This single chromosome 10 integration was corroborated at the level of individual reads. When the quality-filtered reads were mapped to the hybrid reference, the reads associated with the cassette contig aligned predominantly to that contig, to chromosome 10, and to chromosome 11. The chromosome 10 alignments corresponded to junction-spanning reads: in the Integrated Genomics Viewer (IGV), these reads aligned to unique chromosome 10 sequence up to the junction coordinates and became soft-clipped beyond them, with the clipped portions forming supplementary alignments to the cassette contig. The chromosome 11 alignments instead read short, homology-arm-sized blocks that clustered at the H11 locus and were internal to the array (between cassette copies), rather than forming a continuous unique flank leading to a discrete breakpoint; these therefore represent the H11-derived homology arms carried within the cassette rather than a second integration event. No other locus showed read support consistent with integration. Together with the assembly based analysis, these data establish a single transgene integration site on chromosome 10.

### 3.3. Validation of Cre-Inducible HA-Rabl6a Protein Expression Within Tissues In Vivo

**Figure 3:**
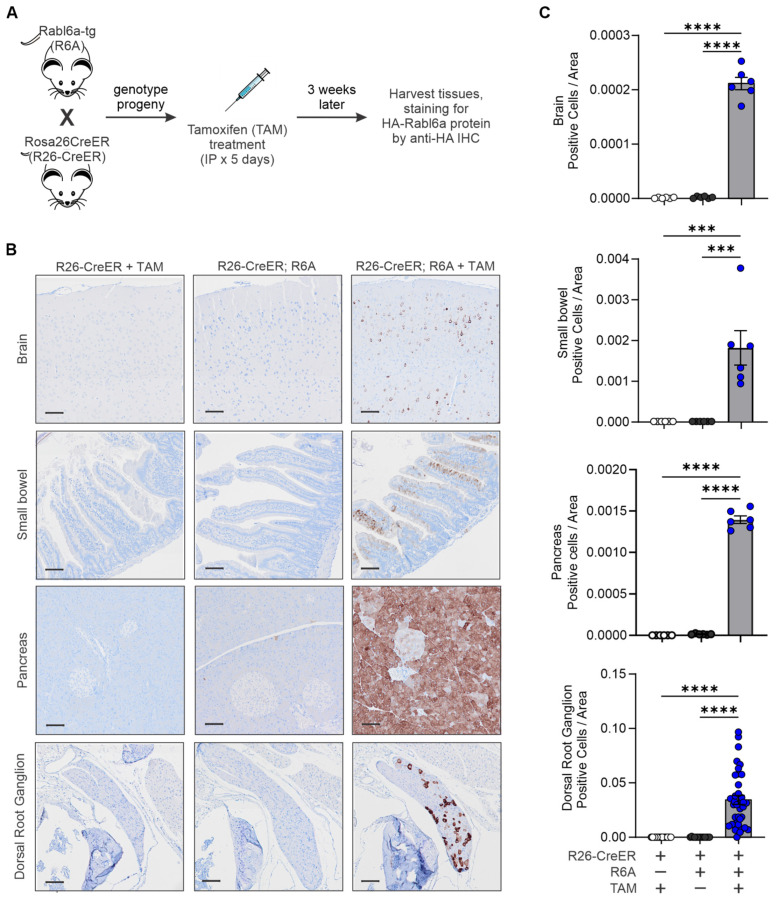
Immunohistochemical validation of Cre-inducible HA-Rabl6a protein expression in vivo. (**A**) Experimental design schematic. Rabl6a-tg (R6A) mice were bred to Rosa26CreER (R26-CreER) mice and offspring treated with tamoxifen (TAM) prior to staining for HA-Rabl6a expression by anti-HA immunohistochemistry (IHC). (**B**) Representative IHC images of HA staining in the brain, small bowel, pancreas, and dorsal root ganglion of the indicated mice treated with or without TAM. Two mice were used per group. (**C**) Quantified HA staining in the different tissues. Each dot graphed for the brain, small bowel, and pancreas represents a quantified image, while each dot represents a separate DRG (multiple DRGs per mouse). Data show significant Cre-inducible expression of HA-Rabl6a expression only in R26-CreER; R6A mice treated with TAM (blue highlighted data points). In contrast, HA staining is essentially absent in tissues from control mice (R26-CreER + TAM or R26-CreER; R6A without TAM). ***, *p* < 0.001; ****, *p* < 0.0001.

Before investigating the effects of increased Rabl6a on MPNST growth, we first verified Cre-inducible expression of the HA-Rabl6a protein in vivo. Various tissues from R26-CreER; R6A mice were examined by immunohistochemical (IHC) staining for the HA tag (Figure 3). No mouse Rabl6a-specific antibodies exist that work under IHC conditions; therefore, expression of the HA-tagged Rabl6a protein was tracked using anti-HA antibodies. As diagrammed in Figure 3A, R26-CreER mice were first crossed with R6A transgenics to generate R26-CreER; R6A progeny and R26-CreER negative controls.

Two mice for each genotype (one of each sex) were treated with TAM for 3 weeks while a cohort of R26-CreER; R6A mice were left untreated to control for Cre expression and the effects of tamoxifen treatment. Several tissues that displayed Cre-inducible expression of HA-Rabl6a protein in TAM-treated R26-CreER; R6A mice were the brain, small bowel, pancreas, and dorsal root ganglion (Figure 3B). Essentially no expression of HA-Rabl6a was observed in control R26-CreER mice treated with TAM or untreated R26-CreER; R6A mice (quantified in Figure 3C). A minimal level of leaky HA-Rabl6a was seen in the pancreas of untreated R26-CreER; R6A mice (Figure 3B,C). No transgene was detected in the liver, lung, skeletal muscle, testes, or ovaries, while a limited number of cells in the gray matter of the spinal cord were positive for HA-Rabl6a (Figure S2). All tissues were stained with H&E and no deviations in normal tissue structure were observed.

Examination of HA-positive tissues from TAM-treated R26-CreER; R6A mice yielded several interesting observations. In the brain, HA-Rabl6a was primarily expressed in the cortical region and hippocampus (Figure 3B,C). Further staining is needed to determine whether the positive cells are astrocytes and/or neurons, although prior analyses of human tissues suggest RABL6A is expressed in both cell types. In the small bowel, around 10–30% of the enterocytes in the villi of the intestine expressed HA-Rabl6a (Figure 3B,C). The greatest staining for HA-Rabl6a was observed in the pancreas, mostly in the acini but also to a lesser degree in the islets, with minor scattered acinar staining in negative control tissue (Figure 3B,C).

Dorsal root ganglion (DRG) cells were intensely positive for HA-Rabl6a (Figure 3B,C). Only DRGs from R26-CreER; R6A mice treated with TAM were positive for HA-Rabl6a with staining in 15–50% of the total nerve cell bodies per DRG. The percentage of HA-Rabl6a positivity within the DRGs inversely correlated with the age of the mouse (Figure S3), where the highest expression was in the youngest mice. The high level of Rabl6a expression in the dorsal root ganglia (DRG) in R26-CreER; R6A mice highlights how R6A mice may be valuable for studying other human pathologies. After a peripheral nerve injury, the DRG can become inflamed and remain dysfunctional, resulting in chronic neuropathic pain [38,39]. Moreover, while much more needs to be learned about Rabl6a molecular functions, it is a Rab-like GTPase and alterations in Rab proteins cause membrane trafficking defects that strongly correlate with neurodegeneration [40]. Thus, while not related to our tumor study, Cre-mediated upregulation of Rabl6a in DRGs and neurons may be useful in studying neuropathic pain and neurodegenerative diseases.

### 3.4. Transgenic Rabl6a Expression Accelerates the Progression of De Novo MPNSTs

**Figure 4:**
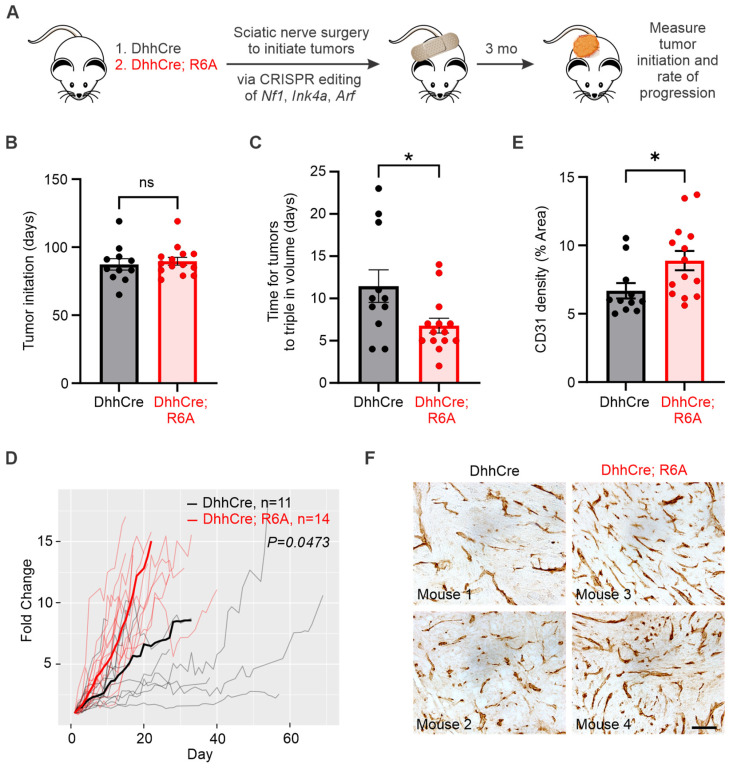
Schwann cell-driven Rabl6a expression enhances the progression, not the initiation, of de novo MPNSTs. (**A**) Schematic of experimental design. De novo MPNSTs were generated by adenoviral CRISPR/Cas9 editing of *Nf1*, *Ink4a*, and *Arf* genes in the sciatic nerve of 11 DhhCre mice and 14 DhhCre; Rabl6a-tg (DhhCre; R6A) mice, yielding tumors approximately 3 months post-surgery. (**B**) Time (days) for tumors to initiate shows identical rates of initiation in DhhCre controls and DhhCre; R6A mice. (**C**) Time (in days) for tumors to triple in volume. DhhCre; R6A tumors grew faster, as shown by a shorter time to triple in size compared to DhhCre control tumors. *, *p* < 0.05. (**D**) Fold change in tumor volume over time shows DhhCre; R6A tumors progressed more quickly than DhhCre tumors. A linear mixed model was used to compare the two progression rates (*DhhCre v DhhCre*; *R6A*, *b* = −0.0457, *SE* = 0.0218, *t* = −2.0963, *p* = 0.0473). (**E**) CD31 density is significantly higher in DhhCre; R6A tumors compared to DhhCre tumors. *, *p* < 0.05. (**F**) Representative images (shown for two different mice for each genotype) of CD31 IHC, scale bar is 100 µm.

The R6A transgenic mouse was created to model RABL6A overexpression in human cancers and directly test its tumor-promoting activity. To study the predicted oncogenic effects of elevated Rabl6a specifically in MPNSTs, we bred R6A animals to DhhCre mice that express Cre recombinase under the Desert hedgehog promoter. This enables targeted expression of Rabl6a in Schwann cells, the presumed cell of origin for MPNSTs, as the Dhh promoter becomes active in Schwann cell progenitors on embryonic day 13.5 [41,42]. De novo MPNSTs were generated by adenoviral CRISPR/Cas9 editing of *Nf1*, *Ink4a*, and *Arf* genes in the sciatic nerve of DhhCre; R6A and control DhhCre mice (Figure 4A). As described previously [35], this approach recapitulates the inactivation of *NF1* that initiates all patient MPNSTs and loss of *CDKN2A* (*INK4a/ARF*) that is seen in 80% or more of these tumors. Mice develop tumors approximately 3 months after sciatic nerve gene editing (Figure 4A). *Rabl6a* transgene expression in each DhhCre; R6A tumor was verified by RT-qPCR using a forward primer specific to the unique HA sequence in the transgene (Figure S4A,B).

Rates of de novo MPNST initiation and progression were compared between DhhCre; R6A and DhhCre mice. Tumor volumes, once they became reliably measurable following CRISPR editing of *Nf1*, *Ink4a*, and *Arf* in the sciatic nerve, were monitored daily with calipers, which is important given the aggressive nature of these tumors. Tumor initiation rates were unaffected by Rabl6a expression despite its expression in Schwann cells prior to tumor onset. Tumors in DhhCre; R6A mice arose on average by day 87 while DhhCre tumors were palpable by day 89 (Figure 4B and Figure S4C). Thus, Rabl6a expression is not sufficient to promote de novo MPNST initiation.

By comparison, transgenic Rabl6a significantly accelerated MPNST progression. This was demonstrated by an approximately 2-fold shorter time for tumors to triple in size in DhhCre; R6A mice relative to DhhCre controls (Figure 4C) and faster kinetics of tumor progression over time in DhhCre; R6A mice (Figure 4D, average fold change can be seen in Figure S4D). Interestingly, there was no difference in the number of Ki67-positive cells per high-powered field between the DhhCre; R6A mice and the DhhCre control mice (Figure S4E,F). The difference in the growth kinetics and lack of difference in Ki67 suggests that Rabl6a promotes MPNST growth through mechanisms other than enhanced proliferation, potentially by increasing angiogenesis, as seen in pancreatic neuroendocrine tumors [43]. In support of this mechanism, increased CD31 positivity was observed in MPNSTs within DhhCre; R6A mice versus DhhCre control mice (Figure 4E,F). CD31 (or PECAM-1) is an endothelial cell marker that quantifies vascular density and angiogenesis in tumors. Together, these data demonstrate for the first time that increased Rabl6a promotes MPNST progression in vivo, which may be driven by enhanced tumor angiogenesis. This result is highly consistent with prior findings from *Rabl6a* knockout mice showing that Rabl6a deficiency reduces both angiogenesis and progression of neuroendocrine tumors [43].

### 3.5. De Novo MPNSTs in a DhhCre Background Display Rhabdomyoblastic Differentiation That Mimics a Subtype of Human MPNSTs

**Figure 5:**
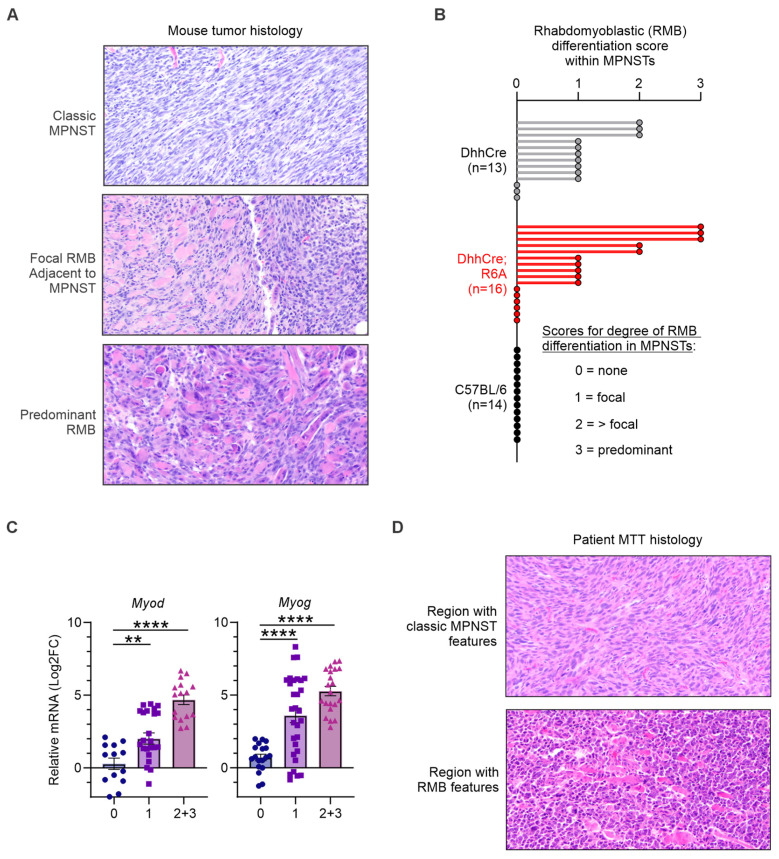
De novo MPNSTs from DhhCre mice exhibit rhabdomyoblastic (RMB) differentiation that mimics malignant Triton tumors (MTTs), an MPNST subtype, in patients. (**A**) Representative H&E images of mouse MPNSTs displaying classic histology, a focal rhabdomyoblastic (RMB) region adjacent to the classic MPNST phenotype, and predominant RMB differentiation. All tumors were confirmed MPNSTs shown at 10X magnification. (**B**) Quantification of RMB differentiation levels within MPNSTs. Text on the left describes each score for the different degrees of RMB differentiation, while each bar in the graph represents an individual mouse MPNST. No RMB was observed in 14 non-DhhCre animals (C57BL/6N). In total, 13 DhhCre tumors and 16 DhhCre; R6A tumors were evaluated. (**C**) Quantification of myoblastic marker expression by RT-qPCR. Significantly increased expression of *Myod* and *Myog* was detected in MPNSTs with focal RMB (level 1) and more than focal RMB (levels 2 and 3) relative to MPNSTs with no RMB differentiation. Tumors scored as levels 2 and 3 were combined due to low sample number for level 3. **, *p* < 0.01, ****, *p* < 0.0001. (**D**) Representative images of patient MPNSTs with an example of typical MPNST histology (top) and of MTT with RMB differentiation (bottom).

Histopathologic analyses were performed on all de novo MPNSTs in the study (Figure 5). Blinded evaluation by two pathologists confirmed the presence of classic MPNST histology for every tumor. Unexpectedly, more than half of the tumors in both DhhCre; R6A and DhhCre mice also displayed varying amounts of rhabdomyoblastic (RMB) differentiation. This unique phenotype, which was absent from non-DhhCre wild-type mice [35,44], was characterized by a mix of malignant Schwann cells (spindle cells arranged in a fascicular architecture with heterogenous “marbled” cellularity) with rhabdomyoblasts, or immature skeletal muscle cells seen as large, often multinucleated, eosinophilic (pink) cells that may exhibit striations. Representative images of this RMB histology are shown in Figure 5A.

The range of RMB differentiation scores for every tumor within each mouse genotype is graphed in Figure 5B. RMB levels were semi-quantified using ordinal scoring as focal (1), more than focal (2), or predominant (3) within the MPNSTs, compared to none (0). Three DhhCre; R6A MPNSTs displayed a predominant RMB differentiation score of three compared to zero for DhhCre tumors, yet more DhhCre; R6A tumors also lacked the RMB phenotype (six of 16 [37%]) relative to DhhCre controls (3 of 13 [23%]). Consistent with the presence of rhabdomyoblastic differentiation, MPNSTs with RMB scores of one to three expressed higher levels of myoblast differentiation markers, *Myod* and *Myog* [45], compared to tumors with classic MPNST histology and an RMB score of 0 (Figure 5C). We attempted to re-examine the rates of tumor growth for RMB-positive versus RMB-negative tumors in each mouse genotype. The analysis suggested that the presence of RMB differentiation did not affect tumor progression, although more animals in each group would be needed to adequately power the comparisons and draw definitive conclusions.

In patients, MPNSTs with regions of RMB differentiation are termed “malignant Triton tumors” (MTTs) [46]. MTTs are a rare subtype (~5–10%) of MPNSTs that express the myogenic markers *Myod* and *Myog* (which are diagnostic for this tumor) [46], have a worse prognosis than traditional MPNSTs [47], and are thought to arise from the transformation of Schwann cells into striated muscle elements [48]. Figure 5D shows representative images of a human MTT, which possesses both classic MPNST histology as well as the same type of RMB differentiation observed in the de novo mouse MPNSTs, including muscle progenitor-like cells with multiple nuclei.

## 4. Discussion

MPNSTs are poorly understood and RABL6A is an under-researched oncoprotein. This study examines the biological importance of elevated murine Rabl6a in MPNST pathogenesis in vivo. Years ago, our lab began investigating human RABL6A in pancreatic adenocarcinoma and neuroendocrine tumors [20,27,43,49], with more recent analyses in MPNSTs [13,17,24,25]. RABL6A function has also been studied in esophageal squamous carcinoma, breast cancer, osteosarcoma, hepatocellular carcinoma, and non-small-cell lung cancer, where its high expression in patient tumors is associated with poor outcomes [18,19,21,22,23]. The cumulative data all point to a tumor-promoting role for RABL6A. However, this is the first study to employ a Rabl6a transgenic (R6A) mouse capable of recapitulating the elevated RABL6A observed in human cancers. Our work establishes the value of this new model for investigating the in vivo role of Rabl6a in MPNSTs, tumor angiogenesis, and other RABL6A-high cancers.

Through Schwann cell-specific expression of Rabl6a in DhhCre; R6A mice, we found that Rabl6a increases tumor vasculature and drives MPNST progression but has no effect on tumor initiation. Specifically, Rabl6a transgenic animals had significantly increased rates of de novo MPNST growth relative to DhhCre controls while tumors arose at identical rates in both animals. Those results agree with previous findings in *Rabl6a* knockout mice, which showed that Rabl6a loss delayed the progression of de novo MPNSTs but had no effect on when tumors formed [24]. Data from human tumors similarly suggest a more important role for RABL6A in promoting disease progression. That is because benign PNFs and ANNUBPs, the precursors to MPNSTs, have quite low levels of RABL6A protein, whereas patient-matched MPNSTs display robust RABL6A expression [13]. One limitation of our model is that Rabl6a-tg expression is turned on early in Schwann cells during embryogenesis such that it is upregulated before the cells undergo transformation to form tumors. To better model the timing of Rabl6a expression seen in human tumors, an inducible Schwann cell-specific Cre model could be used in the future to express Rabl6a after tumor formation.

The DhhCre; R6A mouse represents a robust platform for modeling MPNSTs as the tumors recapitulate the varied morphologies seen in patient MPNSTs. All tumors retained the characteristic histology of MPNSTs with alternating regions of hyper- and hypocellularity, and some areas resemble a herringbone pattern distinct to this disease [4,46]. Yet many MPNSTs in both DhhCre; R6A and DhhCre mice had an interesting histology akin to MTTs, a more deadly subtype of human MPNSTs [46]. Indeed, more than half of tumors in the DhhCre background contained some degree of rhabdomyoblastic (RMB) differentiation, or features of immature skeletal muscle cells. Such cells can form multinucleated cells resembling immature myotubes, and they express myogenic regulatory factors like Myogenin and MyoD, which are markers used to diagnose MTTs in the clinic [46]. This mix of differentiation with regions that mimic rhabdomyosarcoma, along with areas of true MPNSTs, is the hallmark of MTTs [50,51]. Another lab has generated a similar tumor from allografting muscle derived progenitor cells onto the sciatic nerve in SCID mice [52], but to our knowledge our work offers the first immunocompetent de novo mouse model that mimics this subtype of MPNST.

The underlying cause of the RMB/MTT phenotype in our experiment is not known, but there is some relevant literature that can assist in formulating hypotheses about this mechanism. First, our observation is likely Cre-dependent, since the RMB phenotype was absent in non-DhhCre C57BL/6 mice and has not been seen in any prior de novo MPNST experiments [24,44]. Transgenic DhhCre mice have been used previously to genetically inactivate floxed *Nf1* in Schwann cells, resulting in the formation of dermal and plexiform neurofibromas [42]. Those tumors displayed typical neurofibroma histology with no apparent muscle cell differentiation. Our model uniquely relies on surgery to expose the sciatic nerve for adenoviral delivery of CRISPR editing machinery to ultimately induce de novo MPNSTs, a process that unavoidably damages skeletal muscle [35]. Interestingly, while the Desert hedgehog promoter is inactive in healthy skeletal muscle cells, it is turned on in endothelial and Schwann cell populations within injured muscle fibers [53]. During muscle cell healing, Dhh is upregulated and acts as a cell fate determinant, particularly in fibro/adipogenic progenitors (FAPs). This may be particularly relevant to our DhhCre MPNST model since other work suggests that FAPs are the cell of origin for embryonal rhabdomyosarcomas [54]. Whether or not Rabl6a expression enhances the extent of RMB differentiation cannot currently be determined as more animals would be needed to sufficiently power that analysis. Regardless, our data demonstrate that transgenic expression of Rabl6a increased tumor progression in all tumors whether or not they were defined as “classic” MPNSTs or MTTs.

As part of this study, we validated the ability of a more ubiquitous promoter, Rosa26CreER, to enable tamoxifen-induced expression of transgenic Rabl6a in a variety of tissues. Those with the highest levels of HA-Rabl6a were the pancreas, small bowel, brain, and dorsal root ganglia. Rabl6a transgene expression appeared to be highly Cre-dependent and was only observed in tamoxifen-treated R26-CreER; R6A tissues. A small amount of leaky expression was seen in the pancreas of non-tamoxifen-treated R26-CreER; R6A mice, which likely reflects some cells that have tamoxifen-independent basal Cre activity, a documented feature of all R26-CreER mouse lines [55].

Within the R26-CreER; R6A tissues positive for Rabl6a transgene expression, most displayed uneven expression across individual cells. This was less apparent in the pancreas, where robust expression was seen in nearly every cell, consistent with past research that CMV expression is greatest in the exocrine pancreas of transgenic animals [56]. For all other tissues, the absence or presence of Rabl6a expression may be tied to the proliferation of the Cre-positive cells comprising that tissue. In the small bowel, for instance, groups of enterocytes tended to be positive for Rabl6a, possibly all originating from a recombined crypt cell in a villus [57]. Others have shown that not all cells in R26-CreER mice induce Cre recombinase at the same level, with the doses and timing of tamoxifen treatment significantly affecting reporter induction and yielding disparate Cre activity in different cell types [58]. The efficiency of Cre induction could also have been affected by the age of the mice, where 8–20 weeks of age is considered ideal for Cre induction [59]. In support of this possibility, we found optimal DRG expression of Rabl6a in the youngest mice (8 weeks) relative to mice over 20 weeks of age. These data suggest that R26-CreER is not necessarily the ideal Cre animal for use with the Rabl6a-tg model depending on the tissue where expression is desired. However, our experiments with the DhhCre animals were successful and indicate that a tissue-specific Cre or an AAV delivery method could extend the model’s use beyond the pancreas, small bowel, and nervous tissue, where we had robust expression with the R26-CreER model.

In summary, while de novo MPNSTs in DhhCre; R6A mice are shown herein to provide an excellent model for deeper studies of MPNST biology, crosses of the R6A model to different Cre driver mice should be valuable for investigating other RABL6A-high patient tumors. This includes rare malignancies like brain cancers and other sarcomas besides MPNSTs, such as osteosarcoma, as well as common tumors like breast cancer [17,18,19,20,21,22,23].

## 5. Conclusions

RABL6A is overexpressed in many cancers and is often associated with worse patient outcomes, yet a transgenic animal model that mimics its upregulation in tissues has been lacking. The Rabl6a-tg mouse described herein addresses that need, providing a novel animal model for studying RABL6A-high human tumors. Through specific expression in Schwann cells, we demonstrated that Rabl6a is a bona fide driver of MPNST progression in vivo. This finding provides biological insight into key mechanisms of MPNST pathogenesis, which is important for both justifying and guiding new strategies for more targeted and effective therapies. Moreover, the MTT phenotype of many MPNSTs arising in the DhhCre background offers a valuable opportunity to study the biology of that rare disease subtype.

## Data Availability

The authors are happy to share this mouse model with other researchers upon request. Inquiries can be directed to the corresponding author.

## Supplementary Materials

The following supporting information can be downloaded at: https://www.mdpi.com/article/10.3390/cancers18142230/s1. Figure S1: Identification of Rabl6a transgenic mice by genotyping. Figure S2: Additional tissues stained by IHC to assess HA-Rabl6a protein expression in R26-CreER; R6A mice. Figure S3: Declining expression of HA-Rabl6a in dorsal root ganglion (DRG) cells with increasing mouse age. Figure S4: HA-Rabl6a transgene expression and individual mouse tumor growth curves in DhhCre; R6A double transgenic mice. Figure S5: Original Western blot images used to generate Figure 1 and original gel images used to generate Figures S1 and S4.

## Abbreviations

The following abbreviations are used in this manuscript.

ANNUBP	Atypical Neurofibromatous Neoplasm of Uncertain Biological Potential
*CDKN2A*	Cyclin-Dependent Kinase Inhibitor 2A
DhhCre	Desert Hedgehog Cre
DRG	Dorsal Root Ganglion
H&E	Hematoxylin and Eosin
IHC	Immunohistochemistry
MEFs	Mouse Embryonic Fibroblasts
MPNST	Malignant Peripheral Nerve Sheath Tumor
MTT	Malignant Triton Tumor
NF1	Neurofibromatosis Type I
*NF1*	Neurofibromin gene
PNF	Plexiform Neurofibroma
R26-CreER	B6.129-Gt(ROSA)26Sortm1(cre/ERT2)Tyj/J
R6A	Rabl6a-tg
RABL6A	RAB, member RAS oncogene family like 6 isoform A
RMB	Rhabdomyoblastic
TAM	Tamoxifen
