# Supplementary material for: Development of a Cre-Inducible Rabl6a Transgenic Mouse Model That Enhances Sarcoma Growth In Vivo
Source: Cancers (Basel). 2026 Jul 11;18(14):2230. doi: 10.3390/cancers18142230 (PMC13407154; doi:10.3390/cancers18142230)

Supplemental Figures

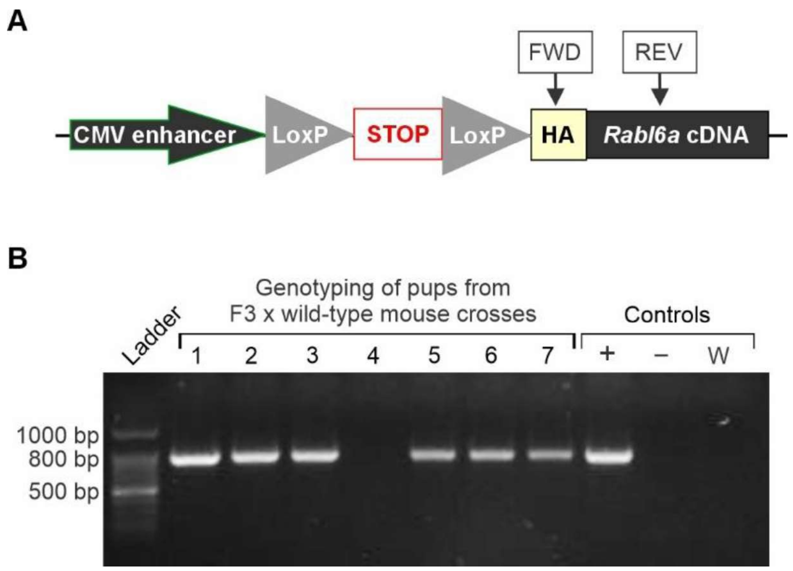

Figure S1 Identification of Rabl6a transgenic mice by genotyping. **(A)** Schematic indicating the location of forward and reverse primers that amplify a single 800 bp HA-Rabl6a DNA product by PCR. (**B)** Example genotyping results example with six mice that are positive for the transgene and one that is negative. Positive control (+), negative control (-), and water control (W) are shown to the right.

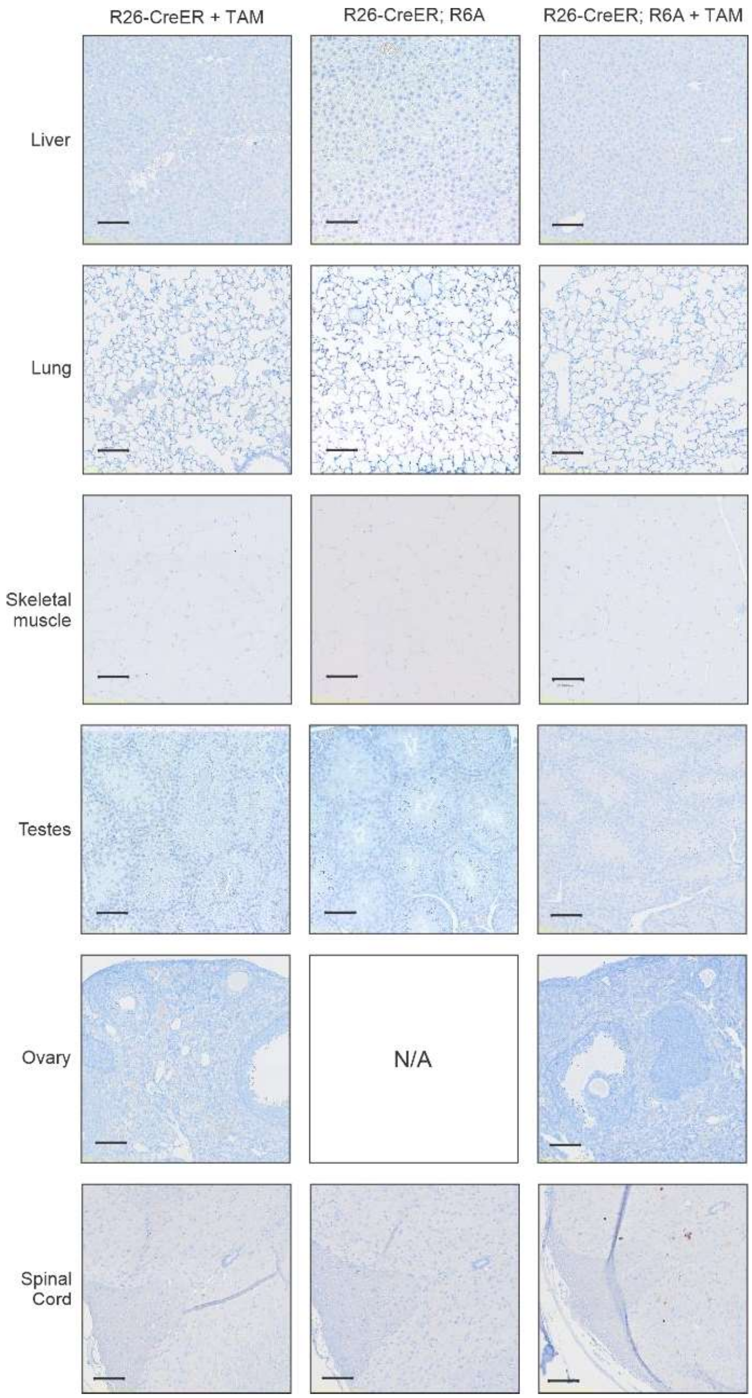

Figure S2 Additional tissues stained by IHC to assess HA-Rabl6a protein expression in R26-CreER; R6A mice. As displayed in the main text Figure 3, HA-Rabl6a (R6A) expression was examined by anti-HA IHC staining in tissues isolated from the offspring of R6A mice crossed with Rosa26CreER (R26-CreER) mice and treated with or without tamoxifen (TAM). Most of the tissues shown here were negative for transgenic R6A protein expression in R26-CreER; R6A + TAM mice. This included the liver, lung, skeletal muscle, testes, and ovary. However, the spinal cord displayed a scattering of positively stained cells. No gross pathology abnormalities were observed in any tissues from the experimental R26-CreER; R6A + TAM mice or control animals. No ovaries were harvested from the R26-CreER; R6A mouse.

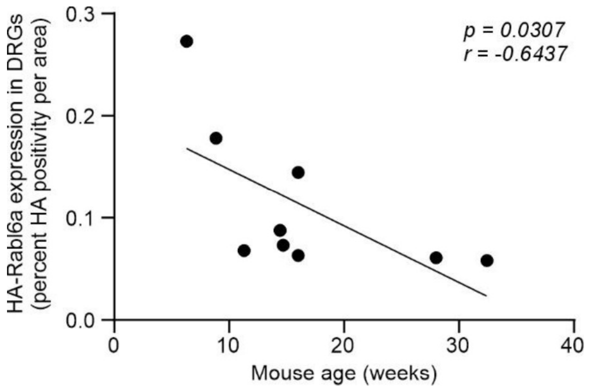

Figure S3 Declining expression of HA-Rabl6a in dorsal root ganglion (DRG) cells with increasing mouse age. There are nine mice (6 female and 3 male) represented ranging in age from 9 weeks to 32 weeks old.

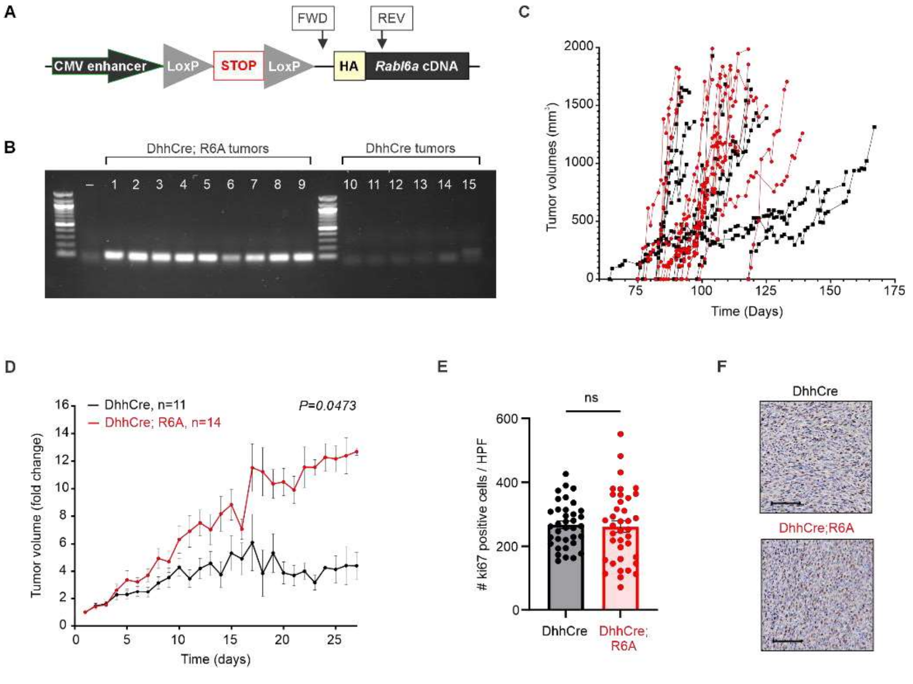

Figure S4 *HA-Rabl6a* transgene expression in DhhCre; R6A double transgenic mice, additional tumor growth data, and Ki67 staining of tumors. **(A)** Location of the primers for qPCR amplification of the HA-Rabl6a cDNA. (**B)** Agarose gel showing selective amplification of the 90 bp HA-Rabl6a product only in tumors from DhhCre; R6A mice (samples 1-9) versus no product in tumors from DhhCre control mice (samples 10-15). The first lane is a negative control (-) cDNA from WT MEFs. (**C**) Spider plot of each animal's tumor volume over time (days). No sex differences were observed within or between groups. (**D)** Average fold change in tumor growth over time for DhhCre; R6A (n=14) versus DhhCre (n=11) tumors. **(E)** No difference in percent of Ki67 positive cells per high powered field (HPF) between DhhCre and DhhCre; R6A tumors. **(F)** Representative images of Ki67 staining in DhhCre tumors (top) and DhhCre; R6A tumors (bottom). Scale bar=100 μm.

Figure S5 Raw images of western blots and gels used in the indicated figures are presented in the following order: Raw images of western blots used for Figures 1C and 1D, Gel images for Rabl6a-tg mouse genotyping used in Figure S1, and Gel images for HA-Rabl6a qPCR used in Figure S4.

Figure 1C, also used for quantification in 1D
Blot #1

Proteins:
Rabl6a ~120 kDa
Gapdh 37 kDa

Sample ID:
1. Human HA.R6A in H1299 cells
2. R6A MEFs
3. R6A + TAM MEFs
4. R26-CreER, R6A MEFs
5. R26-CreER, R6A + TAM MEFs
6. R26-CreER MEFs
7. R26-CreER + TAM MEFs

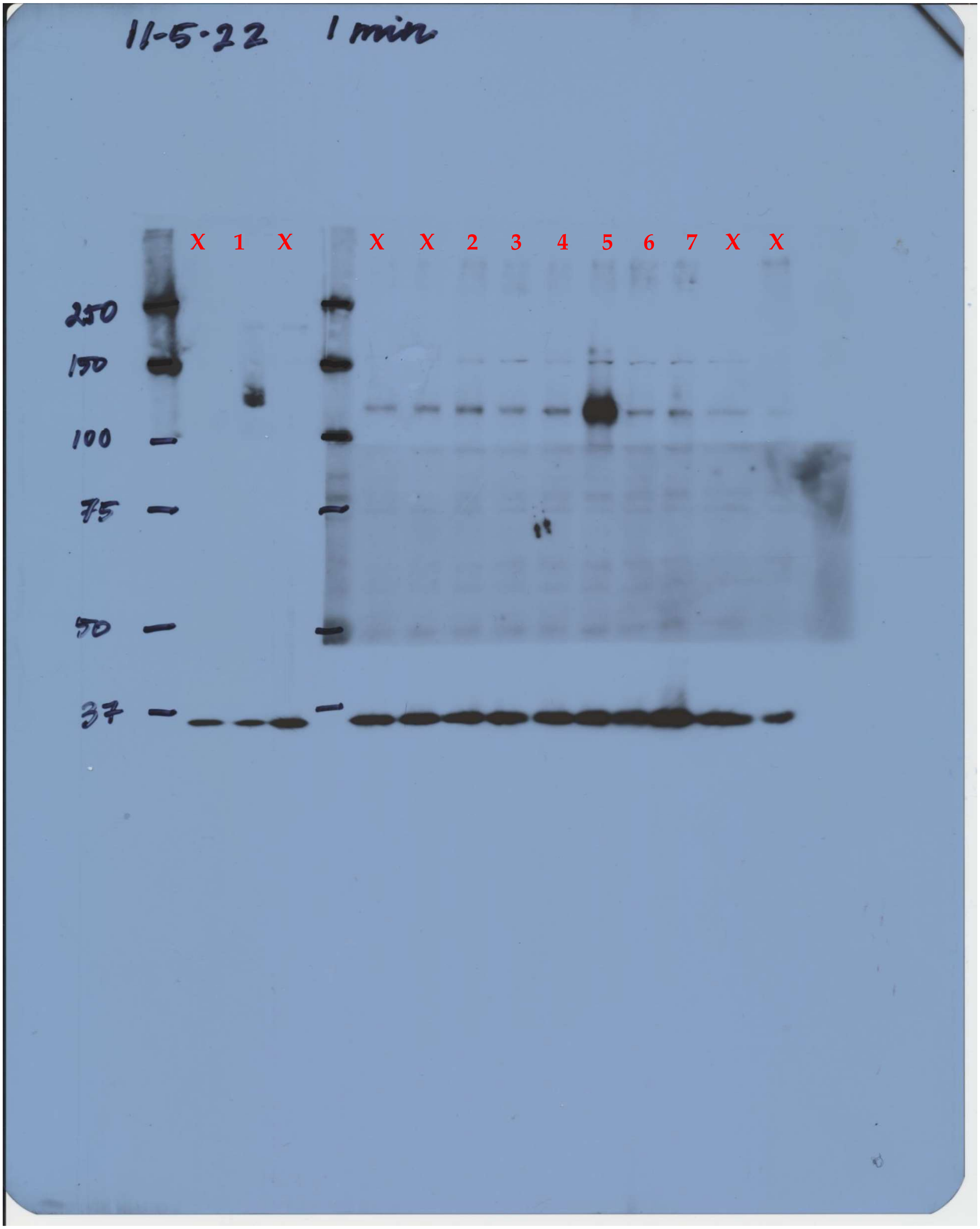

Figure 1D:
Blot #2 used for quantification

Proteins:
Rabl6a ~120 kDa
Gapdh 37 kDa

Sample ID:
1. R26-CreER MEFs
2. R26-CreER + TAM MEFs
3. R6A MEFs
4. R6A + TAM MEFs
5. R26-CreER, R6A MEFs
6. R26-CreER, R6A + TAM MEFs

X X X X 1 2 3 4 5 6

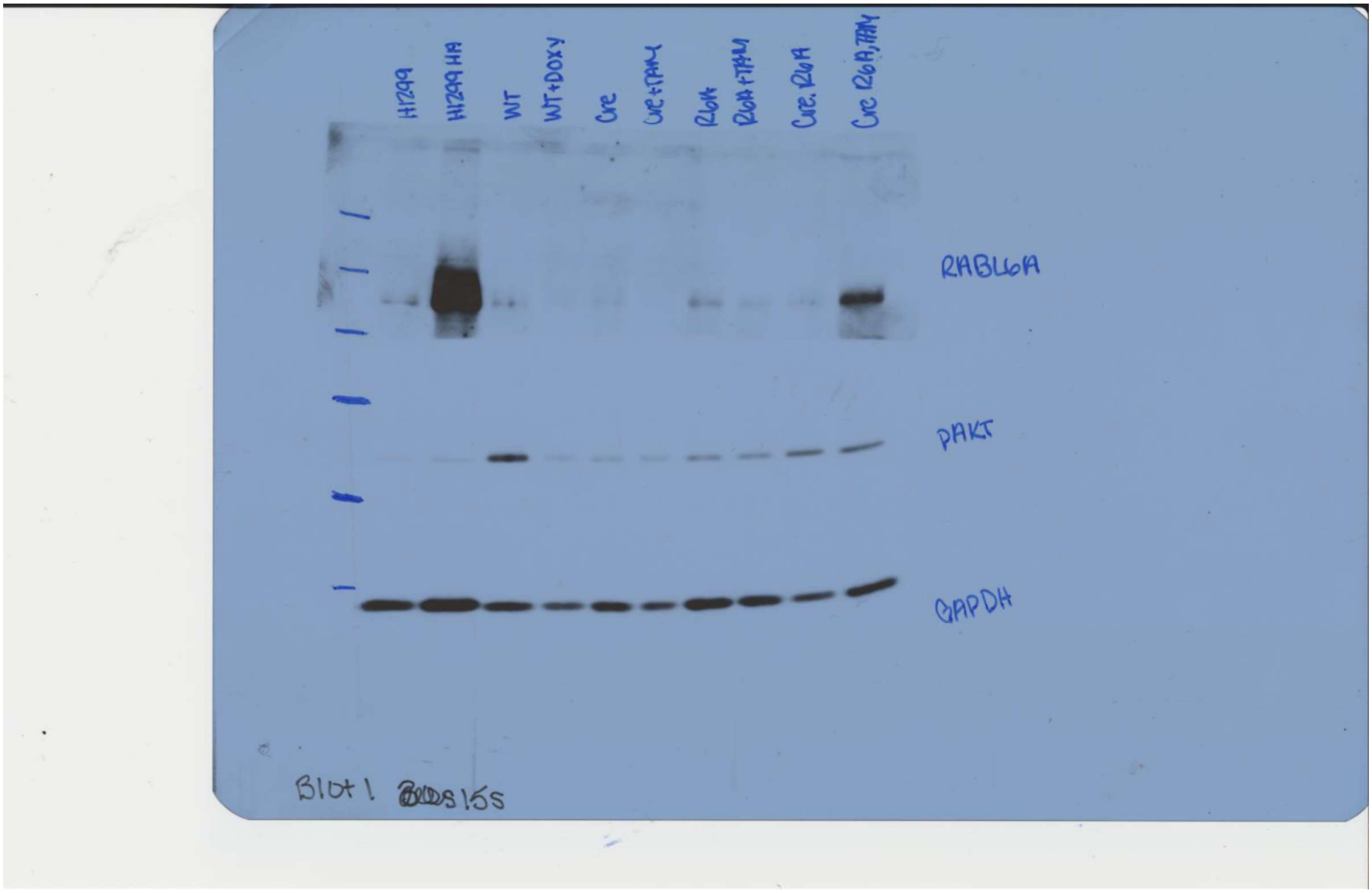

Figure 1D:
Blot #3 used for quantification

Proteins:
Rabl6a ~120 kDa
Gapdh 37 kDa

Sample ID:
1. R6A MEFs
2. R6A + TAM MEFs
3. R26-CreER, R6A MEFs
4. R26-CreER, R6A + TAM MEFs

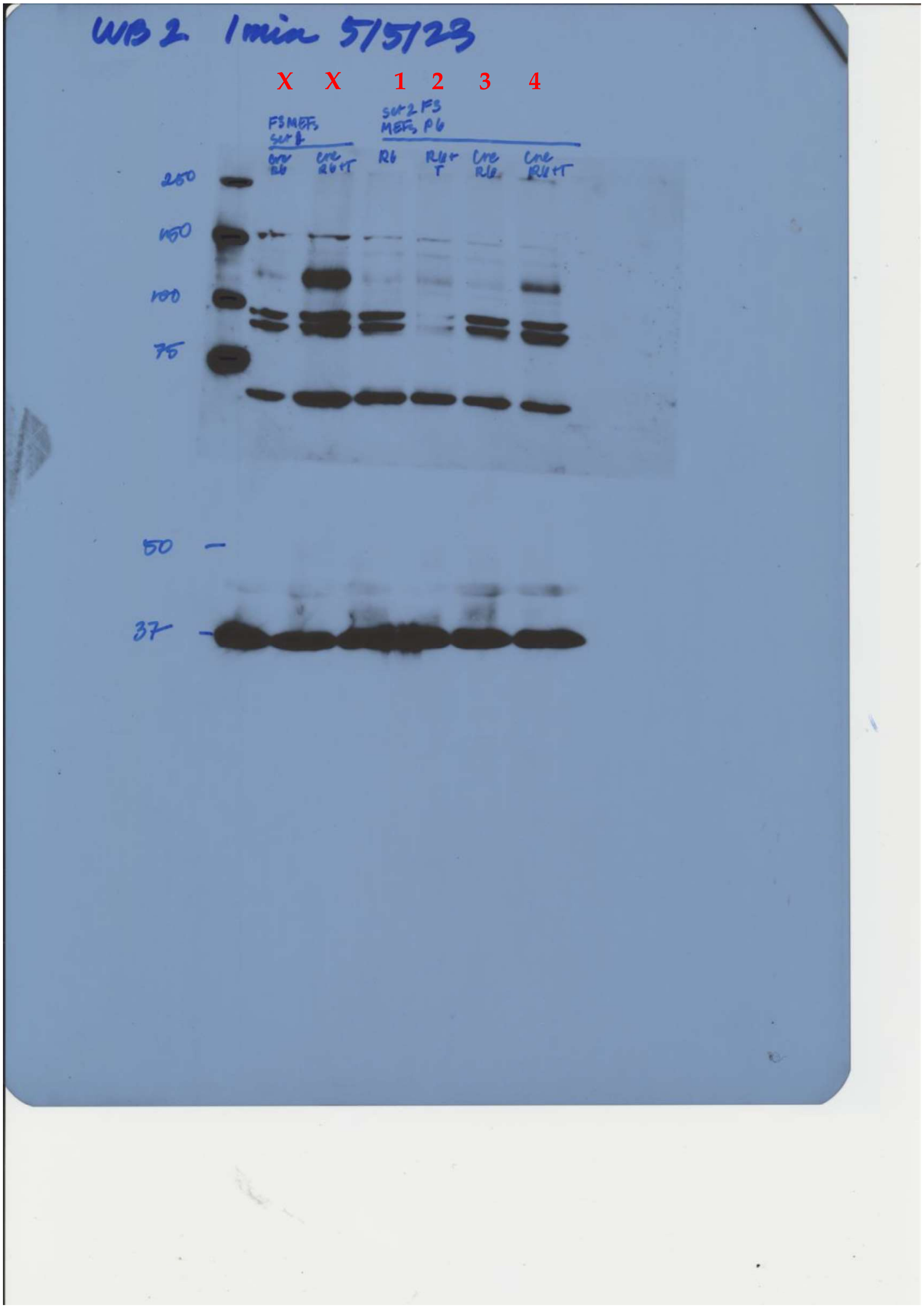

Figure S1
Rabl6a-tg mouse genotyping

Sample ID:

1. DNA from pup 1
2. DNA from pup 2
3. DNA from pup 3
4. DNA from pup 4
5. DNA from pup 5
6. DNA from pup 6
7. DNA from pup 7
8. Positive control
9. Negative control
10. Water control

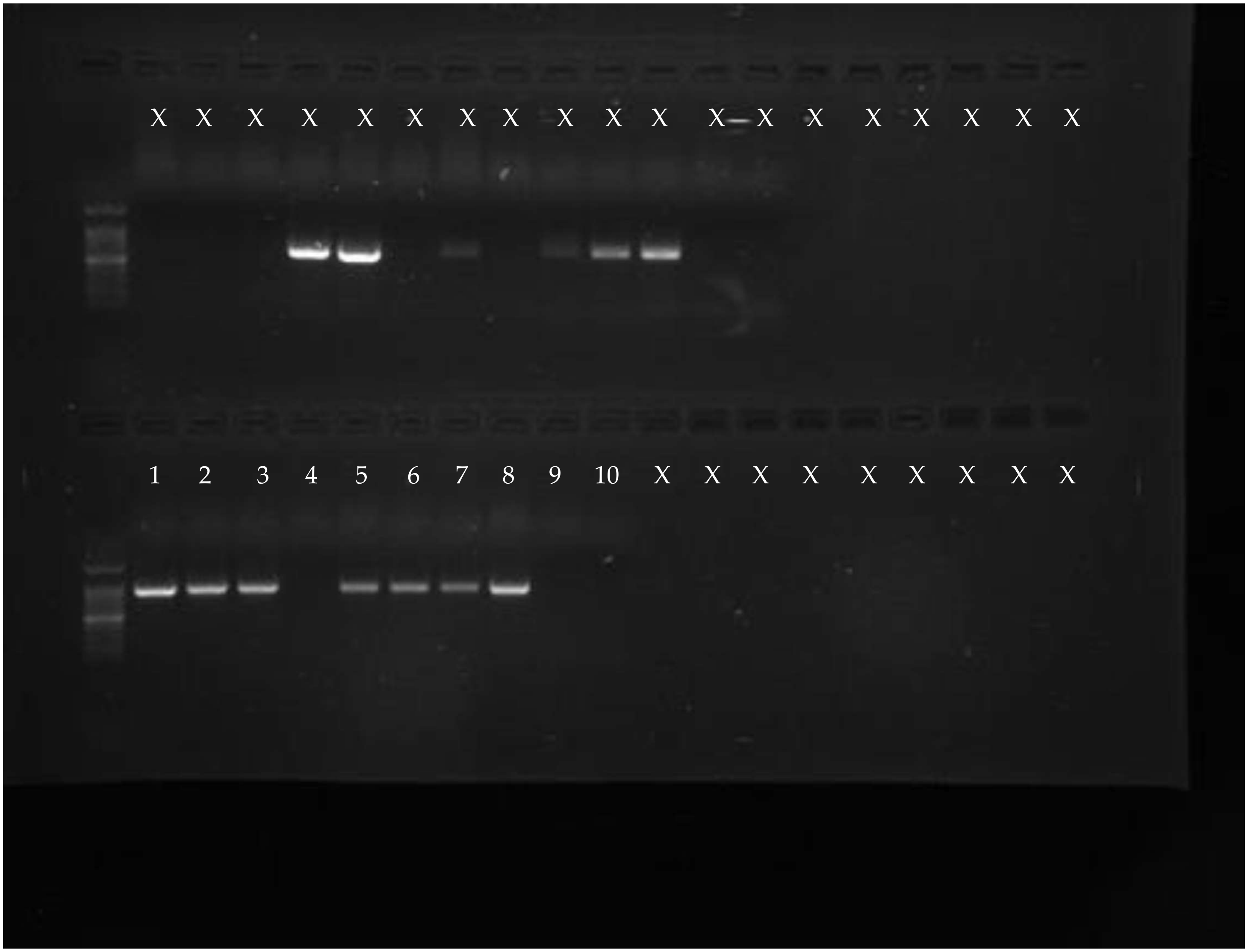

Figure S4
HA-Rabl6a qPCR

Sample ID:
1. Negative control
2. DhhCre;R6A tumor
3. DhhCre;R6A tumor
4. DhhCre;R6A tumor
5. DhhCre;R6A tumor
6. DhhCre;R6A tumor
7. DhhCre;R6A tumor
8. DhhCre;R6A tumor
9. DhhCre;R6A tumor
10. DhhCre;R6A tumor
11. DhhCre tumor
12. DhhCre tumor
13. DhhCre tumor
14. DhhCre tumor
15. DhhCre tumor

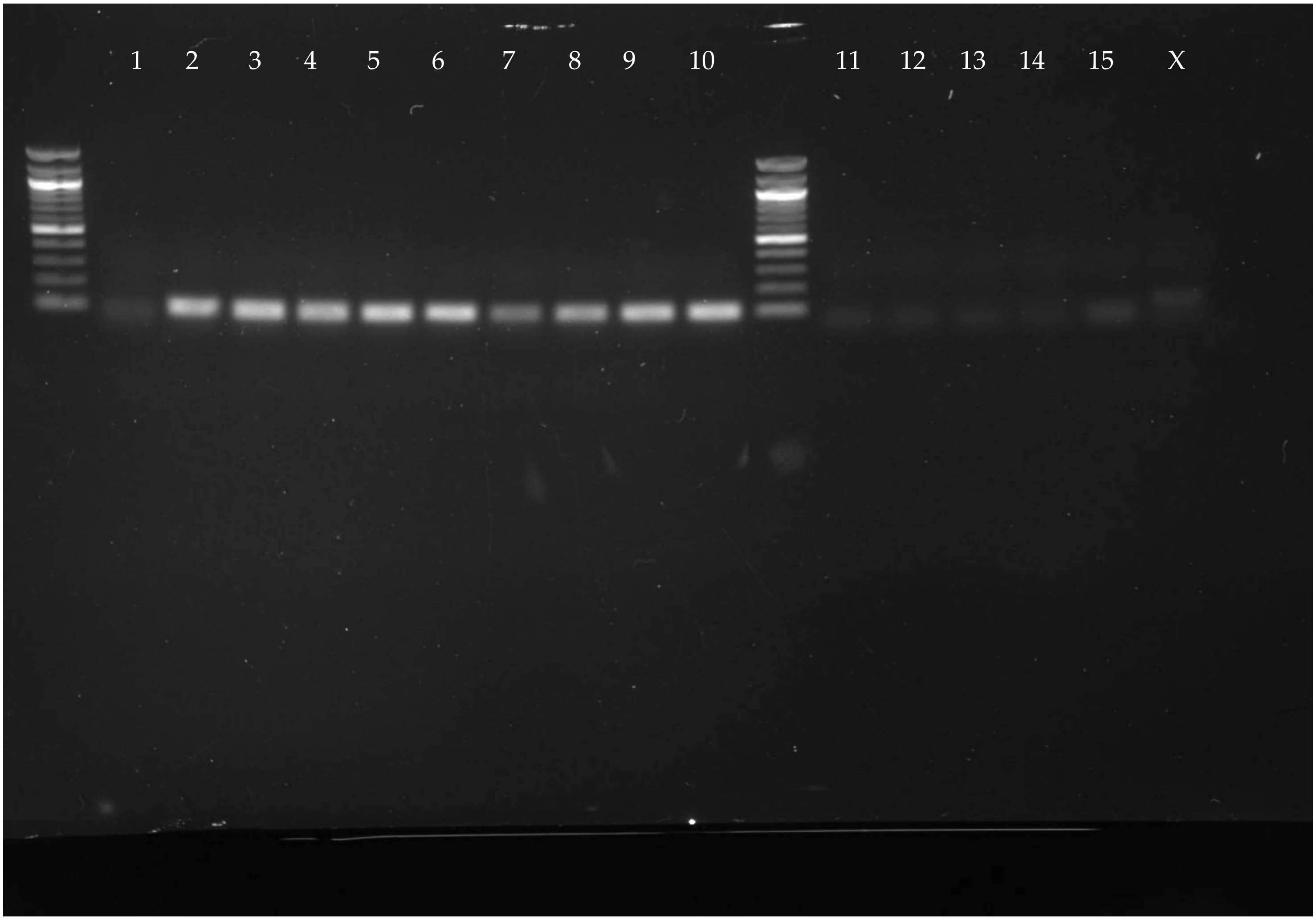

Supplement: Supplementary file 1 [file cancers-18-02230-s001.zip › cancers-4310681-supplementary.pdf]
